# Epigenetic impact of infection on carcinogenesis: mechanisms and applications

**DOI:** 10.1186/s13073-016-0267-2

**Published:** 2016-01-28

**Authors:** Naoko Hattori, Toshikazu Ushijima

**Affiliations:** Division of Epigenomics, National Cancer Center Research Institute, 5-1-1 Tsukiji, Chuo-ku, Tokyo 104-0045 Japan

## Abstract

Viral and bacterial infections are involved in the development of human cancers, such as liver, nasopharyngeal, cervical, head and neck, and gastric cancers. Aberrant DNA methylation is frequently present in these cancers, and some of the aberrantly methylated genes are causally involved in cancer development and progression. Notably, aberrant DNA methylation can be present even in non-cancerous or precancerous tissues, and its levels correlate with the risk of cancer development, producing a so-called ‘epigenetic field for cancerization’. Mechanistically, most viral or bacterial infections induce DNA methylation indirectly via chronic inflammation, but recent studies have indicated that some viruses have direct effects on the epigenetic machinery of host cells. From a translational viewpoint, a recent multicenter prospective cohort study demonstrated that assessment of the extent of alterations in DNA methylation in non-cancerous tissues can be used to predict cancer risk. Furthermore, suppression of aberrant DNA methylation was shown to be a useful strategy for cancer prevention in an animal model. Here, we review the involvement of aberrant DNA methylation in various types of infection-associated cancers, along with individual induction mechanisms, and we discuss the application of these findings for cancer prevention, diagnosis, and therapy.

## Background

Viral and bacterial infections are strongly related to human carcinogenesis, and viral infections account for 10–15 % of human cancers worldwide [1, 2]. Infection-associated cancers (Table 1) include hepatocellular carcinomas (HCCs) induced by the hepatitis B virus (HBV) and hepatitis C virus (HCV) [3–5]; lymphomas, nasopharyngeal cancers (NPCs), and gastric cancers associated with the Epstein-Barr virus (EBV) [6, 7]; cervical and head and neck squamous cell cancers (HNSCCs) induced by human papillomavirus (HPV) [8, 9]; Merkel cell carcinoma associated with Merkel cell polyomavirus (MCPyV) [10, 11]; and gastric cancers induced by *Helicobacter pylori* [12]. The carcinogenic mechanisms of these infection-associated cancers have been extensively investigated, focusing on the effects of viral and bacterial infections and the resultant inflammation on cell proliferation, cell signaling, and genetic alterations [1].

**Table 1:** Infection-associated cancers and aberrant DNA methylation

Bacterium or virus	Cancer type	Tumor-suppressor genes methylated	Direct or indirect effect	Factors involved in induction of aberrant methylation
*Helicobacter pylori*	Gastric cancer	*p16*, *LOX* [39], *miR-124a* [47], *miR-34b/c* [48], *ANGPTL4* [46], *FHL1* [44]	Indirect	Inflammation
Epstein-Barr virus	Gastric cancer	*p73* [72], *TFF1* [73]	Direct and/or indirect	Latent membrane proteins
	Nasopharyngeal cancer	*DLC1* [79], *DAPK* [77], *p15*, *p16*, *RASSF1A*, *TSLC1* [78]	Unknown	Unknown
	Burkitt’s lymphoma	*BIM*, *PRDM1* [84]	Unknown	Unknown
Hepatitis B virus	Hepatocellular carcinoma	*p16* [97], *p21*, *CDH1* [18], *SOCS1* [103], *RASSF1A*, *GSTP1* [98]	Direct and/or indirect	HBx and inflammation
Hepatitis C virus	Hepatocellular carcinoma	*p16* [97], *RASSF1A*, *GSTP1* [98], *RIZ1* [103]	Indirect	Inflammation
Human papillomavirus	Head and neck squamous cell carcinoma	*p16*, *CDH1*, *RARβ* [115], *MGMT* [114], *DAPK* [113], *DCC*, *GALR1*, *GALR2*	Unknown	Unknown
	Cervical cancer	*p16*, *FHIT*, *GSTP1*, *MGMT* [120], *MAL* [122], *TSLC1* [123]	Unknown	Unknown
Merkel cell polyomavirus	Merkel cell carcinoma	*RASSF1A* [161]	Unknown	Unknown

*HBx* HBV encoded protein X

In addition to these effects, induction of epigenetic alterations is now regarded as one of the most important mechanisms mediating the effect of viral or bacterial infections on cancer development. The first reports of an association between viral infections and DNA methylation date back to the 1970s; these reports demonstrated an increase in global 5-methylcytosine in cells transformed by adenovirus and polyomavirus [13, 14]. In the 2000s, aberrant DNA methylation of tumor-suppressor genes was detected first in EBV-infection-associated cancers [15] and then in gastric mucosae of individuals with *H. pylori* infection [16, 17]. To date, many studies have demonstrated a relationship between viral or bacterial infections and aberrant DNA methylation [18–20].

Chronologically, aberrant DNA methylation can already have accumulated in non-cancerous or precancerous tissues, producing an ‘epigenetic field defect’ or ‘epigenetic field for cancerization’ [21]. The epigenetic field for cancerization is characterized by accumulation of aberrant methylation of various genes in a tissue without clonal lesions, and by the correlation between the ‘severity’ of a field and cancer risk [21]. The clinical relevance of this concept has recently been demonstrated by a multicenter prospective cohort study to predict risk of metachronous gastric cancer [22].

Mechanistically, aberrant DNA methylation can be induced directly by a component(s) of an infectious agent, as recently shown for EBV [19, 23, 24]. Alternatively and more commonly, aberrant DNA methylation can be induced by chronic inflammation, as robustly shown for *H. pylori* and hepatitis viruses [25, 26]. In addition, a recent study suggested that chronic inflammation could induce histone modification changes more frequently and much earlier than aberrant DNA methylation, and that some of the aberrant histone modifications can serve as a signal for aberrant DNA methylation [27].

In this review, we first summarize the effect of *H. pylori* infection, whose role in induction of aberrant DNA methylation and gastric carcinogenesis has been intensively studied, and introduce the mechanisms of how *H. pylori* infection induces aberrant DNA methylation. Then, we introduce several viral infections that induce aberrant epigenetic alterations, especially DNA methylation, and discuss the mechanisms involved. Finally, we discuss the applications of infection-induced epigenetic alterations for cancer prevention, diagnosis, and therapy.

## *Helicobacter pylori* in gastric cancers

The vast majority of gastric cancer cases worldwide are induced by *H. pylori* infection. It is an archetypal cancer in which chronic inflammation and epigenetic alterations are interconnected. The mechanisms by which *H. pylori* infection induces aberrant DNA methylation have been investigated in gastric cancers, cell lines, and animal models [25, 28]. The epigenetic nature of gastric cancer was recently reported [29].

### Association among *H. pylori* infection, epigenetic alterations, and gastric cancer

Gastric cancer is one of the most common malignancies worldwide, especially in Asia and some European countries [30]. The major risk factor is persistent *H. pylori* infection [31]; risk is elevated 2.2- to 21-fold by *H. pylori* infection [12, 32, 33]. In some Asian countries, nearly all gastric cancer patients have a history of *H. pylori* infection [2]. *H. pylori* is a Gram-negative bacterium [34, 35] and is thought to be transmitted orally within families during early childhood owing to poor hygiene. Few bacteria can survive in the stomach because of its low pH maintained by the production of gastric acids; however, *H. pylori* can survive for decades because of its production of urease, which neutralizes its immediate environment [36]. It induces chronic gastritis characterized by persistent infiltration of neutrophils and mononuclear cells, and gastric atrophy [37]. Gastric atrophy is also a strong risk factor for gastric cancer (hazard ratio = 14.09 (95 % confidence interval (CI) = 7.03–28.26)) [38].

Deep involvement of aberrant DNA methylation in human gastric cancers had been suggested by the fact that tumor-suppressor genes, such as *CDH1*, *p16*, and *hMLH1*, were inactivated more frequently by aberrant DNA methylation of their promoter CpG islands than by genetic alterations [39]. More recently, integrated analysis of DNA methylation and genetic alterations in gastric cancer has revealed that genes involved in cancer-related pathways were more frequently affected by DNA methylation than by genetic alterations [29, 40]. Furthermore, recent exome and whole-genome analyses of gastric cancers have revealed new mutated driver genes, such as *ARID1A*, *FAT4*, and *RHOA*, but the incidences of mutations were 14 %, at the most, among the cancers analyzed [41, 42]. Importantly, a significant number of cancers have few mutations; according to a report by Wang et al. [41], 5 of 100 cancers had no mutations, and 22 cancers had only one mutation. These reports support the major role of DNA methylation in gastric cancer.

The link between *H. pylori* infection and DNA methylation in gastric mucosae was first discussed in two contradictory reports in 2003. Chan et al. [43] demonstrated that promoter methylation of the tumor-suppressor gene *CDH1* was more frequent in the gastric mucosae of individuals with *H. pylori* infection than in uninfected individuals. In contrast, Kang et al. [16] did not detect a difference in the number of methylated genes between the gastric mucosae of individuals with and without *H. pylori* infection. A quantitative DNA methylation analysis of passenger genes (defined as those that do not have a causal role in carcinogenesis) [17] later convincingly demonstrated that *H. pylori* infection was associated with increased DNA methylation levels in gastric mucosae. These findings highlight the importance of accurate quantification of DNA methylation and analysis of appropriate genes.

The increased DNA methylation levels in non-cancerous gastric mucosae with *H. pylori* infection were observed in various but specific genes, including a small number of tumor-suppressor genes, such as *p16*, *ANGPTL4*, and *FHL1*, and a large number of passenger genes [44–46]. In addition to protein-coding genes, microRNA genes, including *miR-124a* and *miR-34b/c*, were also aberrantly methylated in non-cancerous gastric mucosae infected with *H. pylori* [47, 48]. Importantly, the levels of DNA methylation of various methylated genes were relatively consistent and correlated with the risk of gastric cancer development [17, 49].

To investigate the cell types with aberrant methylation of these genes, normal gastric epithelial cells were purified from an animal model, and the presence of aberrant DNA methylation in these gastric epithelial cells was shown [25]. Three positions within the stomach (antrum, middle body, and upper body) had increased methylation levels in individuals with high risk of gastric cancers compared with corresponding positions in the stomach in individuals with low risk [49].

Therefore, aberrant DNA methylation of various specific genes is accumulated in normal gastric epithelial cells in various positions within the stomach of individuals with high risk of gastric cancers, and an ‘epigenetic field defect’ or an ‘epigenetic field for cancerization’, which is an area or even an entire tissue predisposed to cancer development, is produced [21]. Generally, the presence of a cancerization field has been known for decades, but it has been explained by mutation accumulation [50]. Now, based on the findings in gastric cancer [21], accumulation of epigenetic alterations in non-cancerous or precancerous tissue has been shown to be important. The concept is likely to be expanded to several types of other infection-associated cancers, and has great value as a risk marker, as discussed later.

### Mechanisms of induction of aberrant DNA methylation by *H. pylori* infection

From the viewpoint of *H. pylori* infection-induced gastric carcinogenesis, most research has traditionally focused on cell proliferation, induction of genetic instability and mutations, and activation of cellular signaling [51–54]. For example, in the Mongolian gerbil animal model (*Meriones unguiculatus*), in which *H. pylori* infection markedly promotes gastric carcinogenesis by a mutagen such as *N*-methyl-*N*-nitrosourea [55], chronic inflammation due to *H. pylori* infection was shown to induce increased cell proliferation, and this may cause further accumulation of mutations [56]. However, induction of aberrant DNA methylation can be even more important as described above.

**Fig. 1:**
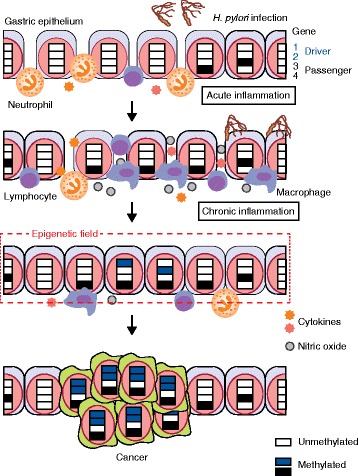
Mechanisms of induction of aberrant DNA methylation by *H. pylori* infection. Acute inflammation following infection by *H. pylori* develops into chronic inflammation characterized by the transition of neutrophil infiltration to that of lymphocytes and macrophages. Chronic inflammation signals, including cytokines such as IL-1β and TNF-α and/or nitric oxide production, are associated with the induction of aberrant DNA methylation. Aberrant DNA methylation is induced both in driver genes (schematically represented by genes 1 and 2) that are causally involved in gastric cancer development and in passenger genes (genes 3 and 4) that are methylated in association with gastric carcinogenesis in normal-appearing tissues. Driver genes are methylated only at very low levels (shown in *blue*), showing that such events are present only in a very small fraction of cells, whereas many passenger genes are methylated at high levels (shown in *black*), showing that their methylation is present in a large fraction of cells. The accumulation of aberrant DNA methylation in normal-appearing tissues produces an ‘epigenetic field for cancerization’, which is an area of tissue or an entire tissue without clonal growth but predisposed to cancer development

Aberrant DNA methylation can potentially be induced by two mechanisms: directly by a component of *H. pylori*, such as DNA methyltransferase, being injected into gastric epithelial cells through a bacterial type IV secretion system [57]; or indirectly due to inflammation triggered by *H. pylori* infection. To clarify which mechanism is important, Niwa et al. [25] used Mongolian gerbils, in which aberrant DNA methylation was induced by *H. pylori* infection in a manner similar to that observed in humans. They treated gerbils infected with *H. pylori* with an immunosuppressant, cyclosporine A, and found that the induction of aberrant DNA methylation was strongly suppressed, whereas *H. pylori* colonization itself was not affected or was even augmented [25]. In addition, one week after *H. pylori* eradication, when no *H. pylori* remained in the stomach but inflammation still persisted, aberrant DNA methylation continued to be induced. These data showed that inflammation triggered by *H. pylori* infection, but not by *H. pylori* itself, is involved in induction of aberrant DNA methylation (Fig. 1).

The next questions are what types of inflammation are involved in DNA methylation induction and what cytokines or molecules mediate the signal from the inflammation. In gerbils, repeated administration of high concentrations of ethanol or salt (NaCl) induced persistent severe inflammation accompanied by strong induction of cell proliferation, but did not result in induction of aberrant DNA methylation [28]. *H. pylori* infection induces chronic inflammation, characterized by a transition of inflammatory cell types from polymorphonuclear cells (mainly neutrophils) to mononuclear cells (lymphocytes and macrophages), with some remnant neutrophils [58]. In contrast, prolonged treatment with ethanol or salt induced repeated acute inflammation, characterized by persistent neutrophil infiltration [28]. This indicates that a specific type of inflammation, possibly characterized by mononuclear cell infiltration, is required for induction of aberrant DNA methylation.

Regarding the responsible cytokines or molecules, increased expression of *Cxcl2*, *Il1b*, and *Tnf* (which encode cytokines) and *Nos2* (which encodes nitric oxide (NO) synthase) were present in gastric mucosae of gerbils with *H. pylori* infection, but not in those of gerbils exposed to ethanol- or salt-induced inflammation [42]. The potential involvement of *IL1B* in human gastric cancer is further evidenced by the association between polymorphisms in the *IL1B* promoter and gastric cancer susceptibility, especially among individuals with *H. pylori* infection [59, 60], although *Il1b*-deficient mice were resistant to *H. pylori*-induced gastric cancers [61]. *IL1B* promoter polymorphisms were also associated with the presence of the CpG island methylator phenotype, a distinct phenotype with frequent aberrant DNA methylation of multiple CpG islands, in gastric cancers [62]. Also, treatment of gastric cancer cell lines (TMK-1, MKN-74, and MKN-7) with IL-1*β* has been reported to have induced the methylation of tumor-suppressor gene *CDH1*, based on a conventional methylation-specific PCR [62, 63].

NO, whose production is enhanced by an *H. pylori* extract [64] and IL-1β [65], was reported to upregulate the enzyme activity of DNA methyltransferases (DNMTs) without affecting mRNA expression levels [65]. However, the enhancement of DNMT activity by NO has not been confirmed since then. In addition, no changes in the mRNA level of genes encoding DNMTs have been observed in human or gerbil stomachs infected with *H. pylori* [25, 66] and in mouse colonic epithelial cells exposed to chronic inflammation [67]. Therefore, the molecules involved in the addition or maintenance of a methyl group are unlikely to be affected, and we suggest that factors that protect DNA from methylation, such as TET proteins, are likely to be affected by chronic inflammation, possibly via signals from macrophages, such as IL-1β, TNF-α, or NO (Fig. 1).

## EBV and cancer

EBV was the first virus detected in human neoplastic cells (in a Burkitt’s lymphoma cell line), in 1964 [68], and is involved in several tumor types. It was recently shown to have a direct effect on induction of aberrant DNA methylation based on an infection system of recombinant EBV in vitro [19, 23, 24].

### Association among EBV infection, epigenetic alterations, and cancer

EBV infection is epidemiologically associated with development of human tumors, such as Burkitt’s lymphoma, Hodgkin’s lymphoma, peripheral natural killer/T-cell lymphoma, smooth muscle tumor, NPCs, and gastric cancer [7]. EBV, a gamma-herpes virus consisting of double-stranded DNA, maintains itself as an episomal circular DNA in the nuclei of infected cells without the production of viral particles, and it is not integrated into the host genome [69, 70]. Although more than 90 % of the world population is infected with EBV before adolescence and become lifelong virus carriers, malignant neoplasms develop in a limited number of carriers.

Approximately 10 % of all gastric cancer cases are EBV-associated, and monoclonal growth of EBV-infected gastric epithelial cells is detected in gastric mucosae [71]. Aberrant DNA methylation was observed more frequently in EBV^+^ cancers than in EBV^-^ cancers [15], and hypermethylation of several specific genes, such as *p73* and *TFF1*, has also been reported [72, 73]. Genome-wide effects of EBV on DNA methylation were revealed by Matsusaka et al. [74], and gastric cancers were categorized into three distinct groups, EBV^-^ and low methylation, EBV^-^ and high methylation, and EBV^+^ and extensively high methylation.

In nasopharyngeal cancer, 70–90 % of the differentiated type is associated with EBV infection [75], and premalignant lesions of the nasopharyngeal epithelium are EBV^+^, suggesting the occurrence of EBV infection at an early step in carcinogenesis [76]. In primary cancers, aberrant DNA methylation has been observed at tumor-suppressor genes, including *DAPK1*, *DLC1*, *p15*, *p16*, and *RASSF1A* [77–79]. Significant association has been observed between the levels of promoter methylation of *RASSF1A* and *TSLC1* tumor-suppressor genes and the quantity of EBV DNA detected in cancer tissues and in adjacent and distant non-cancerous tissues [78].

In lymphoma, EBV is present in over 95 %, 5–10 %, and 3–40 % of endemic, sporadic, and HIV-associated Burkitt’s lymphoma cases, respectively [80], in approximately 40 % of Hodgkin’s lymphoma cases [81], and in 2–9 % of diffuse large B-cell lymphoma cases [82]. Aberrant DNA methylation of tumor-suppressor genes, including *BIM* and *PRDM1*, was detected in EBV^+^ Burkitt’s lymphoma cases but not in EBV^-^ cases [83, 84]. Hansen et al. [85] identified large-scale hypomethylated blocks, which encompassed several Gb or were at least longer than 1 Mb, in EBV-transformed B cells by a genome-wide analysis, suggesting that at the early stage of EBV-associated carcinogenesis, global hypomethylation occurs first, leading to genome instability and eventually to the induction of aberrant DNA methylation.

### Mechanisms of induction of aberrant DNA methylation by EBV

**Fig. 2:**
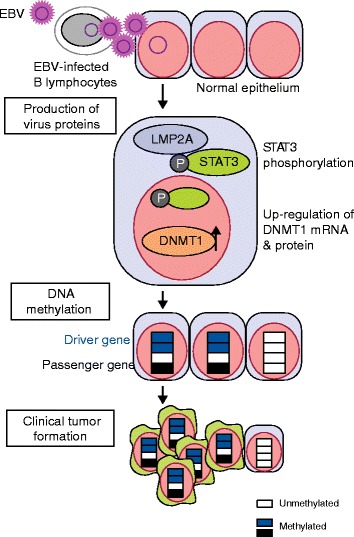
Molecular mechanisms of induction of aberrant DNA methylation by Epstein-Barr virus (*EBV*) infection. In gastric epithelial cells, EBV is transferred to normal epithelial cells from EBV-infected B lymphocytes. EBV produces multiple viral proteins, including latent membrane protein 2A (LMP2A), which activates STAT3. STAT3 induces increased expression of DNMT1, leading to upregulation at the mRNA and protein levels. Upregulation of DNMT1 by STAT3 induces aberrant DNA methylation of multiple genes, including both driver (shown in *blue*) and passenger (shown in *black*) genes

The causal role of EBV infection in inducing high levels of DNA methylation was confirmed by forcing EBV expression in a gastric cancer cell line and observing acquisition of new DNA methylation within 18 weeks [19]. Introduction of latent membrane protein 1 (LMP1), a viral oncoprotein from EBV, into a breast cancer cell line (MCF-7) activated DNMT1, DNMT3A, and DNMT3B, and induced methylation-silencing of tumor-suppressor gene *CDH1* [86]. LMP1 directly activated the *DNMT1* promoter via the JNK-AP1 pathway [87]. Latent membrane protein 2A (LMP2A) also induced *DNMT1* overexpression via STAT3 activation [88], which led to methylation-silencing of tumor-suppressor gene *PTEN*. In contrast with epithelial cells, EBV infection of germinal center B cells, the presumptive progenitors of Hodgkin’s lymphoma, down-regulated DNMT1 and DNMT3B via LMP1, whereas DNMT3A was upregulated at the mRNA and protein levels [89]. Taken together, EBV infection is considered to induce aberrant DNA methylation by its direct effect, namely via dysregulation of DNMTs (Fig. 2).

## HBV and HCV and hepatocellular carcinoma

Infections by HBV and HCV induce chronic hepatitis, and are the major cause of HCC. Involvement of aberrant DNA methylation has been suggested by the increase of aberrantly methylated genes during disease progression (from chronic hepatitis to liver cirrhosis and to HCC). A recent report also showed the importance of the immune response in the induction of methylation by HBV or HCV [26].

HBV is a DNA virus and can be integrated into the host genome, leading to virus-related insertional mutagenesis of tumor-related genes [90]. However, it used to be considered that there was no consensus pattern of insertional mutations among HBV-induced HCC samples [3, 91, 92]. Recent whole-genome sequencing analysis of HCCs revealed that, although HBV was frequently integrated into the *TERT* locus, most other frequently mutated genes had incidences of less than 10 % [93, 94]. HCV is an RNA virus and cannot be integrated into the host genome, but HCV core protein interacts with multiple proteins of host cells and these interactions induce host responses [95, 96].

### Association among hepatitis virus infection, aberrant DNA methylation, and hepatocellular carcinoma

Epigenetically, similar to cancers of other tissues, HCCs are characterized by hypomethylation of repetitive sequences, associated with genomic instability, and aberrant DNA methylation of tumor-suppressor genes, such as *RASSF1A*, *p16*, *SFRP1*, *GADD45A*, and *p15* [18, 97, 98]. These epigenetic alterations accumulate during the course of HCC development [99]. For example, decreased methylation at the LINE-1 and satellite 2 repetitive elements was mainly observed when chronic hepatitis and liver cirrhosis progressed to HCC [100], and methylation of *CHFR* and *SYK*, potential tumor-suppressor genes, increased in advanced HCC [101, 102]. These findings suggest that accumulation of aberrant DNA methylation in non-cancerous tissues, or an epigenetic field for cancerization that is predisposed to cancer development, may also be present in HCC, similar to that described above for *H. pylori* infection in gastric cancer.

Importantly, the DNA methylation profile of liver cirrhosis and HCC is dependent on the type of hepatitis virus. Nishida et al. [103] showed that methylated loci were categorized into three groups: i) loci methylated in normal tissues and that showed increased methylation during HCC development; ii) loci methylated in non-cancerous and HCC tissues; and iii) loci methylated more densely and frequently in HCV^+^ HCC than in HBV^+^ and virus-negative HCC. Other analyses of genome-wide DNA methylation data also showed etiology-dependent methylation profiles of cirrhosis and primary HCC [104, 105].

### Mechanisms of induction of aberrant DNA methylation by HBV and HCV

**Fig. 3:**
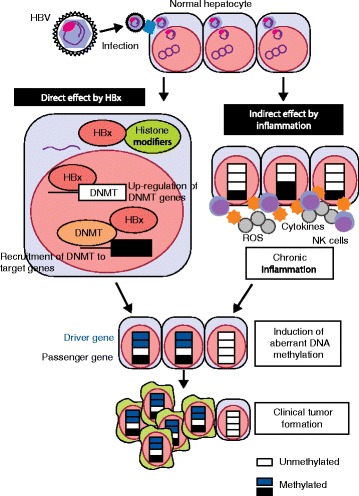
Molecular mechanisms of induction of aberrant DNA methylation by hepatitis B virus (*HBV*). Two mechanisms of HBV-induced DNA methylation have been proposed: a direct effect via hepatitis B virus protein X (HBx) and an indirect effect via chronic inflammation. In the direct mechanism, after HBV infection of hepatocytes, viral DNA is transferred into the nucleus and transcribed. HBx is translated using the host machinery, transported back to the nucleus, and involved in upregulation of *DNMT* genes and recruitment of DNMTs to target genes. In the indirect mechanism, chronic inflammation triggered by HBV infection induces NK cell accumulation, increased *Ifng* expression, and reactive oxygen species (*ROS*) production. Although the molecular details remain to be elucidated, an NK-cell-dependent innate immune response is important for methylation induction

Aberrant DNA methylation may be induced by direct action of a virus and also indirectly via chronic inflammation due to viral infection (Fig. 3). A direct action is known for an HBV protein, hepatitis B virus protein X (HBx). HBx was found to induce *DNMT* upregulation, leading to DNA methylation of the genes involved in the Ras pathway and angiogenesis [106], and to interact directly with DNMT3A to recruit it to the promoters of *IL-4R*, a gene encoding a cytokine receptor involved in apoptosis, and *MT1F*, a potential tumor-suppressor gene, leading to their methylation-silencing [107].

Chronic inflammation is also involved in the induction of aberrant DNA methylation and thus in the production of an epigenetic field for cancerization, similar to the gastric carcinogenesis associated with *H. pylori* infection (Fig. 3). Okamoto et al. [26] analyzed genome-wide DNA methylation in the livers of immunodeficient mice carrying human hepatocytes infected with HBV or HCV. In both systems, aberrant DNA methylation was induced, and the induction was associated with increased expression of an inflammation-related gene, *Ifng*, produced by NK cells, and reactive oxygen species (ROS) production. When NK cell activity was suppressed by the anti-asialo-GM-1 antibody, the induction of aberrant DNA methylation was suppressed, demonstrating that the NK-cell-dependent innate immune response was important for methylation induction [26].

## Human papillomaviruses and squamous cell carcinomas

Human papillomavirus (HPV) is related to the development of HNSCC and cervical cancers [108]. Although details of a mechanistic link between HPV and aberrant DNA methylation are still very limited, clinical application of aberrant DNA methylation in detection of cervical cancers is now actively being investigated [109].

HPV is a circular, double-stranded DNA virus, and more than 100 unique HPV types are known [110]. Among them, HPV types 16, 18, 31, 33, 35 and 45 are high-risk HPV (hrHPV) [108]; 12.5–25 % of HNSCCs are associated with infection by hrHPV types 16, 33, or 35, and HNSCCs in the oropharynx are particularly strongly associated [110, 111]. Approximately 54 % and 17 % of invasive cervical cancer cases are associated with hrHPV types 16 and 18, respectively [112].

Promoter hypermethylation is considered to be a mechanism of HNSCC progression, although analysis has been limited to a small number of tumor-suppressor genes, such as *p16*, *CDH1*, *RARβ*, *MGMT*, *DAPK*, *DCC*, *GALR1*, and *GALR2* [113–115]. Methylation was more frequently observed in HPV^+^ cells than in HPV^-^ cells, and DNMT3A expression was also higher in HPV^+^ cell lines [116–118]. Methylation levels of LINE-1 repetitive elements were higher in HPV^+^ HNSCC than in HPV^-^ cancer cells [119]. This finding was interesting, considering that most cancer cells are characterized by global hypomethylation, including LINE-1 repetitive elements [100]. The authors [100] suggested that HPV-infected cells attempted to silence the virus by DNA methylation, which might have resulted in increased methylation of LINE-1 repetitive elements [119].

In cervical cancer, increased DNA methylation of tumor-suppressor genes, such as *p16*, *FHIT*, *GSTP1*, *MAL* and *TSLC1*, was observed [120–123]. Some of these genes, such as *CADM1* and *MAL*, were also methylated in cervical intraepithelial neoplasia (CIN), a precancerous lesion associated with hrHPV infections [124, 125], providing the presence of an accumulation of aberrant DNA methylation in non-cancerous tissues, or an epigenetic field for cancerization, which is predisposed to cancer development.

## Implications for cancer prevention, diagnosis and therapy

A common characteristic of infection-associated cancers is the presence of an epigenetic field for cancerization [18–20]. In addition to infection-associated cancers, cancers associated with inflammation due to causes other than infection (inflammation-associated cancer), such as Barrett’s cancer of the esophagus, are also associated with an epigenetic field [126]. Furthermore, prostate and breast cancers, both of which are associated with hormonal signals [21], are also associated with an epigenetic field [127, 128]. In addition, DNA methylation in non-cancerous tissues has been associated with tumor aggressiveness and worse patient outcome [129]. These findings indicate that opportunities for cancer prevention, diagnosis, and therapy are potentially widely applicable. In particular, cancer risk diagnosis is now reaching a level of clinical use. Examples of such applications for clinical management of infection-associated cancers are summarized in Table 2.

**Table 2:** Applications for clinical cancer management

Application	Example
Cancer diagnosis	
Cancer risk	Prediction of metachronous gastric cancer [22]
Cancer detection	Early detection of cervical cancer [109]
Cancer prevention	Suppression of *H. pylori*-induced gastric cancers by 5-aza-2′-deoxycytidine in animal model [151]
Cancer therapy	DNA demethylating agents and histone deacetylase inhibitors (not specific to infection-associated cancers) [155]

### Diagnosis of cancer risk

The extent of aberrant DNA methylation accumulation in non-cancerous tissues, or the ‘severity’ of an epigenetic field, correlates with the risk of cancer development, at least in some cancers, including gastric cancer. A recent multicenter prospective cohort study convincingly demonstrated the clinical utility of this concept [22]. In the study, a gastric mucosal biopsy sample was obtained from 826 patients who had undergone endoscopic resection of a gastric cancer, and DNA methylation levels of three preselected marker genes, *EMX1*, *NKX6-1*, and *miR-124a-3* [47, 130], were measured. After a median follow-up of 2.97 years, the patients with a high methylation level of *miR-124a-3* were shown to develop metachronous gastric cancers with a statistically higher incidence (hazard ratio = 2.3, *p* = 0.042). In addition, several promising DNA methylation markers have been identified through retrospective cohort studies [131–133].

Epigenetic cancer risk diagnosis is expected to achieve cancer risk prediction that is very difficult by other means. This is because the assessed epigenome alteration is considered to reflect a patient’s life history, including exposure to environmental carcinogenic factors and how strongly the sampled tissue responded to the carcinogens. Also, DNA methylation levels can be measured precisely and therefore have a methodological advantage. The use of the extent or ‘severity’ of the epigenetic field as a cancer risk marker is expected to be applicable to various types of cancers.

### Early detection of cancers

In HPV-associated cervical cancers, DNA methylation markers have been found to be useful for triage of hrHPV-positive women as a tool for screening [134]. Several methylation biomarkers were able to distinguish cervical samples with intraepithelial neoplasia grade 2 or 3 (CIN2 or 3) from those with CIN1 or without any intraepithelial lesions or malignancy [135–137]. A recent prospective randomized clinical trial by Verhoef et al. [109] compared methylation of preselected marker genes *MAL* and *miR-124-2* with cytology using self-collected cervicovaginal specimens and showed that methylation triage was at least as sensitive as cytology triage for detection of CIN2 or worse. This large-scale randomized prospective study clearly demonstrates the power of epigenetic analysis in detecting cervical cancer.

In EBV-associated NPC, Hutajulu et al. [138] detected aberrant methylation of four tumor-suppressor genes (*DAPK1*, *DLC1*, *CDH13*, and *CADM1*) in DNA from nasopharyngeal brushing samples of cancer patients, high-risk subjects, and healthy EBV carriers, and also detected DNA methylation of *CDH1*, *DAPK1*, and *p16* in the peripheral blood of NPC patients. These reports indicated that the DNA methylation of these genes might be a useful serological marker for screening of primary and local or regional recurrent NPC [139].

### Targets for cancer prevention

Suppression of accumulation of aberrant DNA methylation or elimination of accumulated methylation is expected to lead to a decreased cancer incidence. This concept has been supported by evidence in genetically engineered animal models for colon tumors [140–142], lung tumors [143], blood cancers [144, 145], and squamous cell carcinomas in the tongue and the esophagus [146]. The mechanism has been explained by the induction of cellular differentiation and impairment of stem cell function by decreased methylation due to reduced expression of *Dnmt1* [141, 145]. Also, administration of a DNA demethylating agent, such as 5-aza-2′-deoxycytidine, suppressed tumorigenesis in animal models for intestinal tumors [147], prostate cancer [148, 149], and breast cancer [150].

The plausibility of this strategy in infection-associated cancers is of broad interest considering the large population affected by these types of cancers. Niwa et al. [151] showed that administration of 5-aza-2′-deoxycytidine could suppress the development of *H. pylori*-induced gastric cancers in Mongolian gerbils. In humans, because epidemiological studies have shown an inverse association between the use of nonsteroidal anti-inflammatory drugs (NSAIDs) and incidence of colorectal cancer disease-related death, NSAIDs are used to prevent colorectal cancers [152]. Multiple mechanisms have been proposed to explain the inverse association, including enhancement of apoptosis of colonic epithelia [153, 154]. Therefore, it seems possible that suppression of induction of epigenetic alterations might be effective in infection-associated cancers.

### Targets for cancer therapy

Regarding cancer therapy, epigenetic drugs have been developed that target DNA methyltransferases and histone modification regulators (deacetylases, methyltransferases, demethylases, and readers). DNA demethylating agents and histone deacetylase inhibitors have already been approved for hematological malignancies [155, 156]. Clinical trials for a broader range of tumors, including solid tumors, are being extensively conducted, and various combinations of different epigenetic drugs, or an epigenetic drug and an anti-cancer drug, are also being attempted. Although the targets of epigenetic drugs do not appear to be specific to infection-associated cancers compared with other cancers, infection-associated cancers may have more targets because infection is a potent inducer of epigenetic alterations, and some of these cancers might be a good subpopulation for epigenetic therapy.

## Conclusions and future directions

The induction of aberrant DNA methylation now appears to be the major mechanism by which viral and bacterial infections in various tissues can induce cancer. Therefore, the remaining crucial question is the molecular mechanism by which viral and bacterial infections induce epigenetic alterations. It is clear that, for multiple types of infections, aberrant DNA methylation is induced via chronic inflammation, but the molecular mechanisms by which chronic inflammation induces aberrant DNA methylation are mostly still unclear. At the same time, some pathogens, such as EBV and HBV, directly interfere with epigenetic regulators. Clarification of these molecular mechanisms will have great value in identifying novel targets for cancer prevention.

An epigenetic field for cancerization is a common characteristic of infection-associated cancers. In addition, cancers associated with chronic inflammation due to causes other than infection also have an epigenetic field [126], because chronic inflammation is a potent inducer of aberrant DNA methylation. Furthermore, the presence of an epigenetic field has been reported for hormone-associated cancers [21]. The direct action of hormones on epigenetic machinery has been suggested [157], and more research in this area is also important.

Epigenetic field cancerization provides a broad range of opportunities for cancer diagnosis, prevention, and therapy. The ‘severity’ of an epigenetic field for cancerization is promising as a cancer risk marker, as evidenced by the multicenter prospective cohort study for metachronous gastric cancer [22]. Epigenetic cancer risk markers are considered to reflect the life history of individuals and thus can be effective because environmental exposure is a major cause of human cancers [158]. To bring the markers identified in retrospective cohort studies into practice, more prospective studies in settings with high clinical value are necessary. Early detection of cancer is also a promising application of epigenetic markers, and comparison of sensitivity and specificity with markers now used in clinical practice is necessary.

The use of an epigenetic field as a target of cancer prevention is also awaited. Given that the usefulness of suppression of aberrant DNA methylation has been shown for various types of tumors in animal models, development of a method with minimal adverse effects appears essential. However, current DNA demethylating agents decitabine and azacytidine are mutagenic [159] and cannot be used for cancer prevention. Meanwhile, suppression of chronic inflammation is expected to have preventative value through multiple mechanisms [153, 154]. Drug repositioning, which uses drugs already shown to be safe, may also become a useful strategy to identify drugs targeting aberrant DNA methylation or an epigenetic field, leading to inhibition of infection-associated carcinogenesis.

Epigenetic therapy is currently approved only for hematological malignancies, and as mentioned earlier its application to solid tumors is actively being investigated [160]. Because epigenetic alterations are induced by infection and the resultant chronic inflammation, it is possible that at least some infection-associated cancers will show a good response to epigenetic therapy. If so, identification of epigenomic signatures in tumors that will respond appears to be a topic worthy of investigation.

Aberrant histone modifications have received relatively limited attention, although they appear to occur more often and much earlier than aberrant DNA methylation in a mouse colitis model [27]. One major reason why only a limited number of studies are available is the technical difficulty of quantifying histone modifications, especially for diagnostic applications. Development of a method for precise measurement of histone modifications would advance the field. From preventative and therapeutic viewpoints, many inhibitors of histone modification enzymes and readers are currently being developed [155], and aberrant histone modification induced by infection may be a promising target.

## Abbreviations

CIN: Cervical intraepithelial neoplasia
DNMT: DNA methyltransferases
EBV: Epstein-Barr virus
HBV: Hepatitis B virus
HBx: Hepatitis B virus protein X
HCC: Hepatocellular carcinoma
HCV: Hepatitis C virus
HNSCC: Head and neck squamous cell cancers
HPV: Human papillomavirus
hrHPV: High-risk HPV
LMP: Latent membrane protein
NO: Nitric oxide
NPC: Nasopharyngeal cancer
NSAID: Nonsteroidal anti-inflammatory drug
ROS: Reactive oxygen species
